# Outcomes of Del Nido and hyperkalemic blood cardioplegia in adult cardiac surgery with prolonged aortic cross-clamp times★

**DOI:** 10.1051/ject/2024029

**Published:** 2024-12-20

**Authors:** Malgorzata Szpytma, Damian Gimpel, Jordan Ross, Richard F. Newland, Gareth Crouch, Gregory D. Rice, Jayme S. Bennetts, Robert A. Baker

**Affiliations:** 1 Cardiothoracic Surgical Unit, Division of Surgery, Flinders Medical Centre Adelaide South Australia Australia; 2 Perfusion Service, Cardiothoracic Surgical Unit, Division of Surgery, Flinders Medical Centre Adelaide South Australia Australia; 3 College of Medicine and Public Health, Flinders University; 4 Quality and Outcomes Unit, Cardiothoracic Surgical Unit, Division of Surgery, Flinders Medical Centre Adelaide South Australia Australia

**Keywords:** Troponin, Del Nido, Cardioplegia, Hyperkalemic

## Abstract

*Background*: The utility and uptake of Del Nido cardioplegia in adult cardiac surgery is rapidly increasing. Cases with prolonged aortic cross-clamp times necessitate multi-dosing however an understanding of safe ischaemic times and definitive guidelines in this domain are lacking. Therefore, this study aimed to assess the safety and efficacy of our DNC strategy by comparing post-operative troponin profiles and clinical outcomes between Del Nido and hyperkalaemic cardioplegia for cases with aortic cross-clamp times of greater than 90 min. *Methods*: A single-centre, retrospective cohort study at Flinders Medical Centre and Flinders Private Hospital of patients undergoing composite cardiac surgery with a cross-clamp time longer than 90 min. Data was prospectively collected from the Flinders Cardiac Surgery Registry from June 2014 to December 2022. A propensity-matched (1:1) analysis was performed comparing patients receiving Del Nido cardioplegia (*n* = 194) to those receiving hyperkalemic blood cardioplegia (*n* = 194). The primary outcome was the postoperative troponin release profile with clinical events reported as secondary outcomes. *Results*: There was no difference in the peak or median troponin at 6, 12 and 72 h nor the number of patients with positive troponin profiles postoperatively between cohorts. There was no difference in clinical outcomes between groups with aortic cross-clamp times of 90 min which remained true in sensitivity analysis extending out to 120 min. The Del Nido cohort received less cardioplegia volume (*p* < 0.001) and were more likely to return to spontaneous rhythm (*p* < 0.002). *Conclusion*: Del Nido cardioplegia for anticipated aortic cross-clamp times of greater than 90 min provided equivocal post-operative troponin profiles and clinical outcomes compared to multidose hyperkalemic blood cardioplegia.

## Introduction

The use of Del Nido Cardioplegia (DNC) as an alternative to hyperkalaemic crystalloid blood cardioplegia (HKB) is an accepted practice in cardiothoracic surgery [1]. The use of single-dose DNC for a cross-clamp time of less than 90 min originates from paediatric cardiac surgery [2, 3]. A single dose of DNC provides satisfactory myocardial protection for approximately 90 min [4]. The use of single-dose DNC in adult cardiac surgery with cross-clamp times of less than 90 min is now supported by randomized control trials including that published by Ad et al. [5]. The recent prospective randomized trial by Garcia-Suarez included 474 patients in different settings of adult cardiac surgery not excluding complex procedures and showed comparable outcomes between HKB and DNC [6]. The outcomes of multi dose DNC and determination of safe ischaemic time in adults and in adults with extended cross-clamp time remain unclear. To date there is no clinical guideline supporting the delivery of DNC in cases with extended bypass times.

Current animal models and trials in explanted hearts have shown superior myocardial function and troponin profiles with single dose compared to multi dose DNC regiments [7, 8]. There is also clear evidence of ischaemic changes inferenced by troponin profiles after 90 min of ischaemic conditions in these cohorts [7, 8]. The existing literature on DNC in adults with cross-clamp times over 90 min is currently limited to small patient cohorts or sub-analyses of larger studies and interchangeably defines aortic cross-clamp time and ischaemic time making direct comparisons challenging [4, 9–16]. Ross et al recently reported our unit’s initial experience of DNC however only a small subset (40 patients) had aortic cross-clamp times greater than 90 min in the DNC group [17], while Willekes et al. have reported a propensity matched study of patients with prolonged aortic cross-clamp times [18].

This study reports a review of the safety of DNC within our practice in patients with cross-clamp time exceeding 90 min. We compared those who received HKB with those who received DNC. The primary aim was to assess safety and efficacy based on post-operative Troponin T profile, with the secondary aim to compare post-operative major adverse cardiac events between the two groups.

## Methods

**Figure 1:**
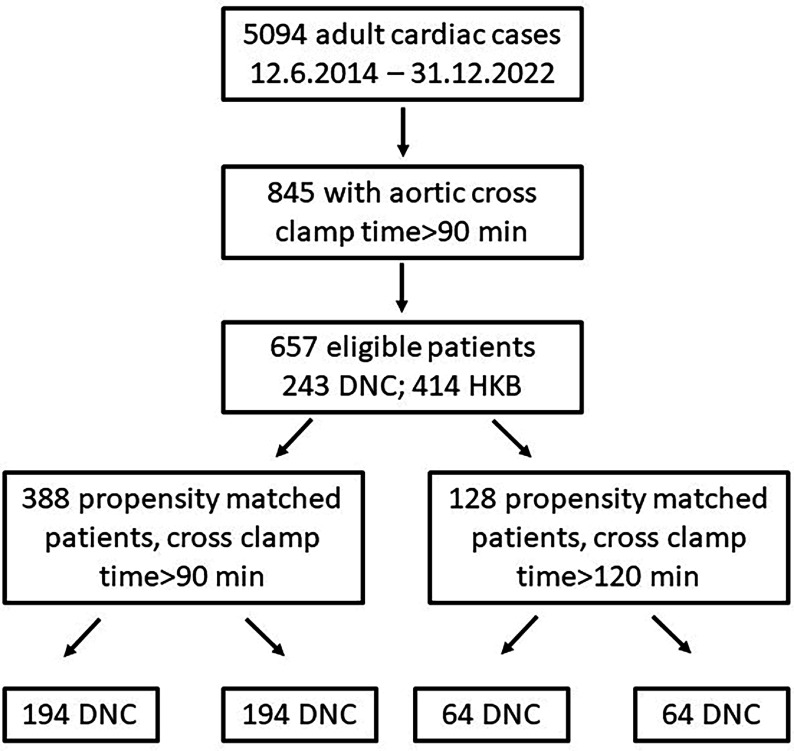
Consort diagram. The 178 patients either had both DNC and HPK cardioplegia (55 cases) or were excluded as the surgeons performing operations did not use both cardioplegic techniques (112 cases). Abbreviations: DNC – Del Nido cardioplegia, HKB – hyperkalemic blood cardioplegia.

This is a single centre, retrospective cohort study including patients who underwent cardiac or aortic surgery, without circulatory arrest, with a cross-clamp time longer than 90 min. Patients from both Flinders Medical Centre and Flinders Private Hospital were included. Patient data was prospectively collected from the Flinders Cardiac Surgery Registry and the Australian New Zealand Collaborative Perfusion Registry from June 2014 to December 2022. During this interval 845 of 5094 patients had aortic cross-clamp times greater than 90 min, of which 188 were excluded from the study (10 no cardioplegia data, 178 as outside of the study period) (Figure 1). Ethics approval for this audit was granted by the Southern Adelaide Clinical Human Research Ethics Committee and the South Australia Local Health Network Office for Research (Quality Registry ID: 2265).

Patients were analysed according to their cardioplegia regimen of either HKB or DNC. DNC was introduced in November 2018. Clinical management, anaesthesia, composition and delivery methods of our DNC and HKB have been previously published [17]. Specifically for DNC, after placement of the aortic cross-clamp, cardioplegic arrest was induced with an antegrade induction dose of 1 L delivered at a flow rate of 200–300 mL/min at 6 °C, targeting aortic root pressures >100 mmHg and less than 150 mmHg. This dose was followed by subsequent 500 mL doses at 60-minute intervals delivered antegrade or retrograde as required. In cases of severe aortic regurgitation, a combination of retrograde and ostial cardioplegia was used. Hyperkalaemic blood cardioplegic arrest was induced with tepid (34 °C) hyperkalaemic blood/crystalloid cardioplegia (induction, 30 mmol/L) at induction and maintained with intermittent doses (maintenance, 15 mmol/L) every 20–30 min. Similar flows and pressures were targeted. In both groups, in addition to the timing of cardioplegia doses, the return of electrical or myocardial contractility was an indication for re-dosing.

Definitions of clinical demography and outcomes were standardised on those reported by the Australian and New Zealand Society of Cardiac and Thoracic Surgeons (ANZSCTS) National Database. Maximum ischemic time was defined as the maximum duration between completion of cardioplegia delivery and either the beginning of the next cardioplegia delivery or reperfusion with cross-clamp removal; our data reflects expected redosing at >90 min in DNC and >30 min in HKB. Mortality was defined as death in a hospital or within 30 days from surgery. Perioperative myocardial infarction was defined as having at least two of the following criteria: ∆Troponin T > 20 ug/L, new regional wall motion abnormalities on echocardiography, Q wave changes on electrocardiogram (ECG). A positive troponin profile was defined as having a troponin value at 72 h following surgery which is the highest troponin measured within 72 h of the index procedure. Acute Kidney Injury (AKI) was defined as postoperative creatinine greater than 150% baseline in accordance with the serum creatinine criteria of the renal Risk, Injury, Failure, Loss of renal function and End-stage renal disease (RIFLE) classification.

The registries meet the Australian Commission on Safety and Quality in Health Care National Operating Principles for Australian Clinical Quality Registries (https://www.safetyandquality.gov.au/publications-and-resources/resource-library/framework-australianclinical-quality-registries). Database managers and staff meet weekly to undertake quality assurance processes. The unit’s general anaesthetic, intraoperative monitoring, cardiopulmonary bypass (CPB), blood conservation and post-operative renal replacement protocols have been previously published [17].

### Statistical analysis

Patients that received DNC were 1:1 propensity-matched without replacement with patients that received HKB, with cross-clamp-times greater than 90 min, providing 194 matched pairs (Figure 1). Preoperative risk factor variables included in propensity matching were age, sex, diabetes, insulin-dependent diabetes, chronic obstructive pulmonary disease, pulmonary hypertension, New York Heart Association classification, left ventricular dysfunction, emergency procedure, cerebrovascular disease, redo procedure, smoking history, elevated preoperative troponin, procedure type, cardiopulmonary bypass time, aortic cross-clamp time, and procedure time. A sensitivity analysis was performed on patients with cross-clamp times greater than 120 min yielding 64 matched pairs.

Stata v 15.1 (StataCorp LLC, Texas) was used for all statistical analyses. Pre-operative, intra-operative and post-operative outcomes were compared between the cohorts. Continuous variables are reported as median with interquartile range and are compared using the Wilcoxon rank-sum test. Categorical variables are reported as a number of patients and group percentage and compared using Fisher’s exact test for variables with binary measures and Pearson’s *χ*^2^ test for categorical variables. A *p*-value of <0.05 was considered statistically significant for all analyses without adjustment for multiple comparisons.

Equivalence in outcome for cardioplegia type was evaluated on the incidence of myocardial infarction, positive troponin profile, and continuous postoperative troponin values at 6, 12 and 72 h, peak, and the 72 h area under the curve, calculated based on the method described by Lakens using the Two One-Sided Tests (TOST) procedure [4]. Continuous troponin values underwent log transformation to approximate normality. The TOST procedure utilised the Fishers exact *Z*-test for proportions. With upper and lower equivalence boundaries defined as Cohen’s *d* ± 0.3, to detect a type I error rate of 0.05 we had a power of 95% for myocardial infarction and 80% for peak postoperative troponin value between primary analysis groups.

## Results

### Patient characteristics

Five thousand and ninety-four adult cardiac cases were performed between 12.6.2014 and 31.12.2022, with 845 having an AXC (aortic cross-clamp) time of greater than 90 min. Six hundred and fifty-seven patients were eligible for inclusion in propensity analyses (see consort diagram in Figure 1), resulting in 194 patients in each group for AXC time > 90 min, and 64 patients in each group for the > 120 min AXC time, with both cohorts demonstrating similar pre-operative characteristics respectively (Tables 1 and 2).

**Table 1:** Patient demographics for aortic cross-clamp time greater than 90 min.

Characteristic	HKB	DNC	*p*-value
*N*	194	194	
Age	64 (52, 72)	64 (49, 73)	0.84
Male	135 (70%)	131 (68%)	0.66
Euroscore II	2 (1, 4)	2 (1, 4)	0.95
BMI	28 (25, 32)	29 (25, 32)	0.90
Diabetic	40 (21%)	42 (22%)	0.80
Insulin dependent	8 (4%)	11 (6%)	0.64
Baseline creatinine (umol/L)	87 (73, 104)	87 (71, 102)	0.91
Dialysis dependent	4 (2%)	3 (2%)	1.00
Pulmonary hypertension	15 (8%)	16 (8%)	1.00
COPD	41 (21%)	45 (23%)	0.62
Smoking history	105 (54%)	97 (50%)	0.42
PVD	8 (4%)	8 (4%)	1.00
Cerebrovascular disease	15 (8%)	16 (8%)	1.00
Redo sternotomy	25 (13%)	23 (12%)	0.76
NYHA			
1	75 (39%)	68 (35%)	0.36
2	64 (33%)	74 (38%)	
3	41 (21%)	32 (16%)	
4	14 (7%)	20 (10%)	
LVEF			
Normal	114 (59%)	110 (57%)	0.71
Mild dysfunction	48 (25%)	56 (29%)	
Moderate dysfunction	24 (12%)	23 (12%)	
Severe dysfunction	8 (4%)	5 (3%)	
Baseline troponin T (ng/L)	49 (25%)	52 (27%)	0.73

Continuous variables are expressed median (IQR), categorical variables are expressed number (%). Abbreviation: HKB – hyperkalaemic blood cardioplegia; DNC – Del Nido cardioplegia; BMI – body mass index; COPD – chronic obstructive pulmonary disease; PVD – peripheral vascular disease; NYHA – New York Heart Association; LVEF – left ventricular ejection fraction.

**Table 2:** Subgroup analysis: Patient demographics for aortic cross-clamp time greater than 120 min.

Characteristic	HKB	DNC	*p*-value
*N*	64	64	
Age	57 (45, 72)	62 (45, 69)	0.83
Male	40 (63%)	46 (72%)	0.26
Euroscore II	2 (1, 5)	2 (1, 5)	0.92
BMI	27 (24, 30)	28 (24, 33)	0.40
Diabetic	11 (17%)	11 (17%)	1.00
Insulin dependent	5 (8%)	2 (3%)	0.44
Baseline creatinine (umol/L)	85 (69, 104)	85 (71, 104)	0.75
Dialysis dependent	1 (2%)	1 (2%)	1.00
Pulmonary hypertension	10 (16%)	9 (14%)	1.00
COPD	19 (30%)	19 (30%)	1.00
Smoking history	33 (52%)	34 (53%)	0.86
PVD	2 (3%)	0 (0%)	0.50
Cerebrovascular disease	7 (11%)	8 (13%)	1.00
Redo sternotomy	10 (16%)	10 (16%)	1.00
NYHA class			
1	26 (41%)	20 (31%)	0.27
2	18 (28%)	27 (42%)	
3	15 (23%)	10 (16%)	
4	5 (8%)	7 (11%)	
LVEF			
Normal	41 (64%)	36 (56%)	0.83
Mild dysfunction	15 (23%)	19 (30%)	
Moderate dysfunction	6 (9%)	7 (11%)	
Severe dysfunction	2 (3%)	2 (3%)	
Baseline troponin T (ng/L)	17 (27%)	18 (28%)	0.84

Continuous variables are expressed median (IQR), categorical variables are expressed number (%). Abbreviation: HKB – hyperkalaemic blood cardioplegia; DNC – Del Nido cardioplegia; BMI – body mass index; COPD – chronic obstructive pulmonary disease; PVD – peripheral vascular disease; NYHA – New York Heart Association; LVEF – left ventricular ejection fraction.

### Primary analysis

Seventy percent of cases in both groups were valve replacement or valve/coronary grafting surgery. There was no significant difference in total AXC time between the two groups. DNC was delivered at a colder temperature, requiring less volume and fewer doses of cardioplegia. Patients receiving HKB were more likely to receive combined antegrade and retrograde cardioplegia delivery. The maximum period of ischemia was significantly greater for DNC compared with HKB (98 (90, 109) vs. 32 (28, 36) *p* < 0.001) and the spontaneous return of rhythm was more likely with the DNC compared to HKB (89% vs. 59%, *p* < 0.001) (Table 3).

**Table 3:** Intraoperative variables for aortic cross-clamp time greater than 90 min.

Variable	HKB	DNC	*p*-value
*N*	194	194	
Procedure type			
CABG	30 (15%)	24 (12%)	0.72
Aortic/Dissection	17 (9%)	21 (11%)	
Other	10 (5%)	14 (7%)	
Valve	90 (46%)	93 (48%)	
Valve/CABG	47 (24%)	42 (22%)	
CPB time (min)	145 (125, 169)	143 (128, 171)	0.98
AXC time (min)	114 (102, 132)	112 (102, 129)	0.45
Total procedure time (min)	269 (229, 307)	266 (232, 320)	0.76
Hemofiltration requirement	7 (4%)	6 (3%)	1.00
Urine output (mL)	400 (220, 700)	400 (200, 750)	0.99
Minimum haemoglobin (g/L)	95 (81, 106)	90 (76, 103)	0.027
Minimum cardioplegia temperature (°C)	31 (30, 31)	5 (5, 6)	<0.001
Total cardioplegia volume delivered (mL)	1885 (1550, 2249)	1009 (1004, 1381)	<0.001
Cardioplegia delivery route			
Antegrade	70 (36%)	146 (75%)	<0.001
Retrograde	0 (0%)	2 (1%)	
Antegrade + Retrograde	124 (64%)	44 (23%)	
Number of cardioplegia doses	6 (5, 7)	1 (1, 2)	<0.001
Spontaneous recovery of rhythm	115 (59%)	170 (89%)	<0.001
Maximum ischemic time (min)	32 (28, 36)	98 (90, 109)	<0.001
Peak creatinine on pump (umol/L)	99 (82, 126)	101 (82, 134)	0.47
Last haemoglobin on pump (g/L)	96 (83, 108)	96 (86, 107)	0.80

Continuous variables are expressed median (IQR), categorical variables are expressed number (%). Abbreviation: Abbreviation: HKB – hyperkalaemic blood cardioplegia; DNC – Del Nido cardioplegia; CABG coronary artery bypass graft; CPB – cardiopulmonary bypass; AXC – aortic cross-clamp.

Clinical outcomes were the same between DNC and HKB for rates of post-operative intra-aortic balloon pump (IABP), myocardial infarction, acute kidney injury, stroke, return to theatre, or mortality (Table 4). The DNC had a higher rate of return to theatre for bleeding (6% vs. 2%).

**Table 4:** Post-operative variables for aortic cross-clamp time greater than 90 min.

Characteristic	HKB	DNC	*p*-value
*N*	194	194	
Mechanical ventilation (h)	17 (8, 25)	18 (8, 27)	0.91
ICU stay (h)	69 (43, 137)	70 (27, 120)	0.60
Hospital stay (day)	9 (7, 13)	10 (7, 14)	0.094
Mortality within 30 days	3 (2%)	8 (4%)	0.22
In hospital mortality	3 (2%)	8 (4%)	0.22
Return to theatre	3 (2%)	12 (6%)	0.017
Stroke	5 (3%)	4 (2%)	1.00
AKI	34 (18%)	39 (20%)	0.52
PRBC	72 (37%)	75 (39%)	0.69
MI	17 (9%)	15 (8%)	0.85
IABP	17 (9%)	10 (5%)	0.23

Continuous variables are expressed median (IQR), categorical variables are expressed number (%). Abbreviations: HKB; hyperkalemic blood cardioplegia; DNC – Del Nido cardioplegia; ICCU – Intensive Critical Care Unit; AKI – acute kidney injury; PRBC – Any red blood cell transfusion; MI – myocardial infarction; IABP – intra-aortic balloon pump

**Figure 2:**
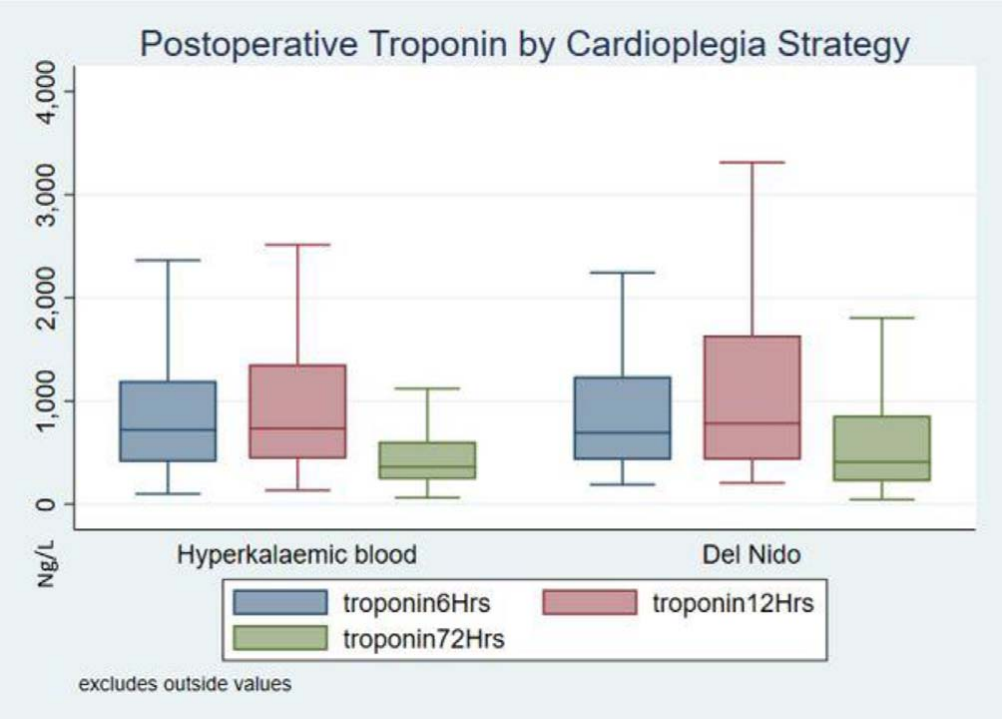
Box and whisker plot for Troponin profile based on cardioplegia strategy for greater than 90-minute ischaemic time. Solid middle bar is the median, top and bottom of box the 75th and 25th percentile, with upper and lower adjacent values.

The median troponin value at time points 6, 12, and 72 h postoperatively, the maximum postoperative troponin, area under the curve nor the number of patients with positive troponin profile showed any difference between the two groups (Table 5 and Figure 2), similarly, there was no difference in time to peak troponin (Table 6). Equivalence testing found DNC to be equivalent to HKC for all troponin measures other than peak troponin (Table 5) (*p* = 0.101).

**Table 5:** Post-operative median troponin profiles for greater than 90 min aortic cross-clamp time.

90-minute aortic cross-clamp	HKB	DNC	*p*-value	Equivalency *p*-value
*N*	194	194		
Troponin T (ng/L)				
6 h	722 (411, 1196)	693 (430, 1237)	0.49	0.04
12 h	735 (441, 1354)	783 (432, 1636)	0.60	0.025
72 h	363 (242, 609)	408 (222, 860)	0.44	0.041
Max	780 (467, 1421)	834 (493, 1853)	0.26	0.081
AUC	37616 (23601, 67166)	39586 (22865, 86359)	0.76	0.021
Positive troponin T rise	8 (5%)	8 (5%)	0.98	<0.001

Continuous variables are expressed median (IQR), categorical variables are expressed number (%). Abbreviations: HKB – hyperkalemic blood cardioplegia; DNC – Del Nido cardioplegia; AUC – area under curve.

**Table 6:** Postoperative peak troponin time as a function of cardioplegia type.

	90-minute aortic cross-clamp		
	HKB	DNC	*p*-value
Patient proportion with peak Troponin T at time interval			
6 h post op	63 (32%)	77 (40%)	0.26
12 h post op	118 (61%)	102 (53%)	
72 h post op	13 (7%)	15 (8%)	

	120-minute aortic cross-lamp		

Patient proportion with peak Troponin T at time interval			
6 h post op	15 (23%)	18 (28%)	0.83
12 h post op	43 (67%)	40 (63%)	
72 h post op	6 (9%)	6 (9%)	

Abbreviation: HKB – hyperkalaemic blood cardioplegia; DNC – Del Nido cardioplegia; post op – postoperative.

### Sensitivity analysis

**Figure 3:**
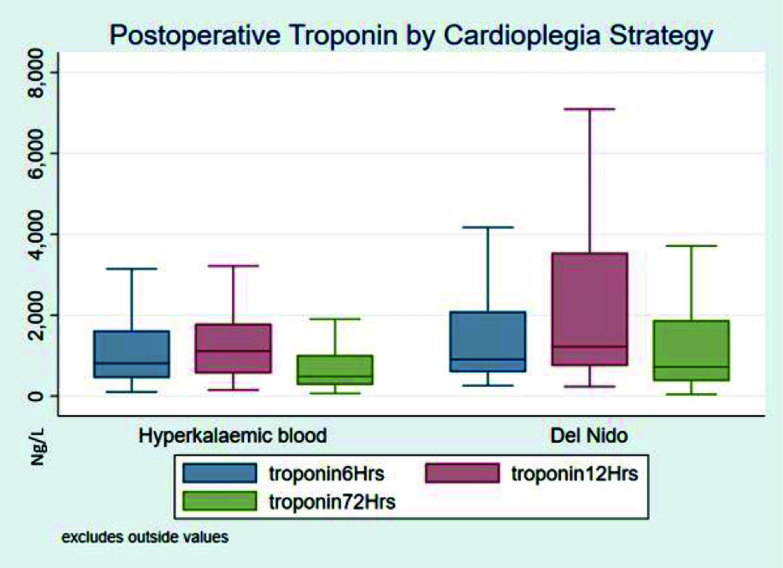
Troponin profile based on cardioplegia strategy for greater than 120-minute ischaemic time. Solid middle bar is the median, top and bottom of box the 75th and 25th percentile, with upper and lower adjacent values.

Similar findings were found for the AXC time > 120-minute subgroup with no differences in preoperative characteristics and clinical outcomes (Tables 2, 7, and 8). Troponin profiling did demonstrate differences, with the 72 h, maximum postoperative troponin value and the area under the release curve being higher in the DNC group (*p* < 0.05, Table 9, Figure 3). The median troponin profile at 6 and 12 h, and the number of patients with positive troponin profile between cohorts were not different (Table 6). Equivalence testing found DNC to be not equivalent in troponin measures other than for positive troponin profile (Table 9).

**Table 7:** Subgroup analysis: Intraoperative variables for aortic cross-clamp time greater than 120 min.

Variable	HKB	DNC	*p*-value
*N*	64	64	
Procedure type			
CABG	5 (8%)	3 (5%)	0.77
Aortic/Dissection	5 (8%)	9 (14%)	
Other	6 (9%)	5 (8%)	
Valve	33 (52%)	31 (48%)	
Valve/CABG	15 (23%)	16 (25%)	
CPB time (min)	185 (160, 210)	190 (166, 217)	0.69
AXC time (min)	146 (129, 165)	143 (130, 173)	0.98
Total procedure time (min)	294 (248, 332)	322 (271, 375)	0.015
Hemofiltration requirement	0 (0%)	4 (6%)	0.12
Fluid output (mL)	400 (200, 750)	460 (250, 1188)	0.35
Minimum haemoglobin (g/L)	91 (77, 103)	91 (76, 101)	0.75
Minimum cardioplegia temperature (°C)	31 (30, 31)	5 (5, 6)	<0.001
Total cardioplegia volume delivered (mL)	2129 (1950, 2486)	1523 (1007, 2009)	<0.001
Cardioplegia delivery route			
Antegrade	30 (47%)	43 (67%)	0.031
Retrograde	0 (0%)	1 (2%)	
Antegrade + Retrograde	34 (53%)	20 (31%)	
Number of cardioplegia doses	7 (6, 8)	2 (1, 4)	<0.001
Spontaneous recovery of rhythm	36 (56%)	56 (89%)	<0.001
Maximum ischemic time (min)	32 (29, 39)	101 (87, 119)	<0.001
Peak creatinine on pump (umol/L)	101 (76, 135)	103 (83, 165)	0.22
Last haemoglobin on pump (g/L)	93 (80, 104)	94 (86, 105)	0.36

Continuous variables are expressed median (IQR), categorical variables are expressed number (%). Abbreviation: Abbreviation: HKB – hyperkalaemic blood cardioplegia; DNC – Del Nido cardioplegia; CABG – coronary artery bypass graft; CPB – cardiopulmonary bypass; AXC – aortic cross-clamp.

**Table 8:** Subgroup analysis: Postoperative variables for aortic cross-clamp time greater than 120 min.

Characteristic	HKB	DNC	*p*-value
*N*	64	64	
Mechanical ventilation (h)	20 (12, 42)	20 (16, 68)	0.40
ICU stay (h)	92 (45, 142)	90 (46, 186)	0.72
Hospital stay (day)	10 (7, 14)	10 (7, 15)	0.49
Mortality within 30 days	2 (3%)	4 (6%)	0.68
In hospital mortality	2 (3%)	4 (6%)	0.68
Return to theatre	1 (2%)	6 (10%)	0.049
Stroke	1 (2%)	2 (3%)	0.62
AKI	11 (17%)	16 (25%)	0.28
PRBC	26 (41%)	29 (47%)	0.49
MI	6 (9%)	11 (17%)	0.20
IABP	8 (13%)	4 (6%)	0.36

Continuous variables are expressed median (IQR), categorical variables are expressed number (%). Abbreviations: HKB – hyperkalemic blood cardioplegia; DNC – Del Nido cardioplegia; ICCU – Intensive Critical Care Unit; AKI – acute kidney injury; PRBC – Any red blood cell transfusion; MI – myocardial infarction; IABP – intra-aortic balloon pump.

**Table 9:** Sub-analysis: Postoperative median troponin profiles for 120-minute aortic cross-clamp time.

	90-minute aortic cross-clamp			
	HKB	DNC	*p*-value	Equivalency *p*-value
*N*	64	64		
Troponin T (ng/L)				
6 h	852 (427, 1455)	907 (593, 2095)	0.14	0.555
12 h	1103 (528, 1795)	1220 (741, 3540)	0.11	0.63
72 h	480 (287, 1015)	720 (372, 1878)	0.030	0.738
Max	1103 (498, 1845)	1406 (807, 3609)	0.019	0.837
AUC	53428 (28151, 101896)	68633 (39815, 19209)	0.042	0.734
Positive troponin T rise	3 (5%)	0	0.074	<0.001

Continuous variables are expressed median (IQR), categorical variables are expressed number (%). Abbreviations: HKB – hyperkalemic blood cardioplegia; DNC – Del Nido cardioplegia; AUC – area under curve.

## Discussion

Del Nido cardioplegia has a well-established safety profile in myocardial ischaemic times of up to 90 min [6], with current literature providing little consensus on management protocols for extended AXC times and optimal reporting of clinical endpoints [13, 19, 20]. Clinical advantages of DNC compared to HKB are thought to be mediated by lidocaine’s inhibition of cardiomyocyte sodium channels, prevention of hypertonic myocardial oedema moderated by mannitol and competitive inhibition of calcium influx by magnesium [7, 21]. Comfortable dosing intervals, and advantages over glycaemic control and reperfusion arrhythmias make it a popular alternative for myocardial protection [6]. The continued reporting of clinical experiences is mandated to build an evidence base upon which practice may evolve.

Our results showed patients receiving DNC had significantly longer ischaemic time compared to the matched HKB group, while demonstrating increased rates of return of spontaneous activity, with no significant differences in clinical outcomes (Table 4). While there was overall equivalence in myocardial injury as inference by post-operative troponin T release (Tables 5 and 6) the optimal timing for DNC re-dosing and clinical endpoints for equivalence remain unclear. Sensitivity analysis of patients with cross-clamp times of greater than 120 min similarly demonstrated no significant differences in clinical outcomes (Table 8), however troponin results were equivocal (Tables 6 and 9).

### Troponin profile

The post-operative release of high-sensitivity troponin T is one measure reflecting the efficacy of myocardial protection and was chosen for this study due to its lower false positive rate compared to other biomarkers such as CKMB. Recent meta-analyses reporting troponin release in the setting of DNC are of limited value and provide little guidance on strategies for cases with extended AXC times [22, 23]. In reporting the troponin T profile for DNC out to 72 h we found equivalence in the median timepoint values and area under the curve between propensity-matched groups in our primary analysis. This is in keeping with the initial single dose experience from our unit suggesting a robustness in the safety profile of our cardioplegia protocol despite DNC having threefold longer ischemic times (98 min vs. 32 min) [17] and the recent report by Willekes et al. [18] where they reported similar findings for patients with extended AXC times. The sensitivity analysis highlights the variation in redosing within this early experience with 25% of patients in the sub-study receiving a single dose of DNC. In this cohort although troponin T profile values within 72 h were not equivalent, equivalence was observed in the incidence of positive troponin T at 72 h. With further ongoing evaluation, the benefit of DNC may be ascertained.

The literature supporting multi-dose DNC is evolving, with reports in early studies of multi-dose DNC showing no difference in post-operative troponin T profile [10, 13], while Willekes [18] showed lower release of Troponin T their functional assessments showed no differences between DNC and HKB. Other studies comparing DNC and HKB have also shown no difference however are limited by design bias [4, 24]. Existing literature reporting lower troponin profiles with DNC has also reflected a concurrent reduction in AXC time [13, 19]. By comparison our study included as a variable in our propensity matching AXC time and this may explain troponin equivalence rather than reduction, even though DNC patients experienced longer ischaemic times. Our early (6 and 12 h) troponin T measurements are consistent with previously reported experiences of non-inferiority for DNC [5, 25]. Ad et al., found lower and earlier peak troponin with DNC; in contrast, Garcia-Suarez et al incorporated more diverse and complex procedures observing an earlier peak (<12 h) with DNC [5, 6]. In contrast to ours, their dosing strategy was a single 1000 mL induction dose mixed with autologous blood (4:1 crystalloid: blood) followed by 500 mL redosing for ischaemic periods >90 min or in patients with spontaneous activity [6].

### Clinical outcomes

Clinical outcomes of DNC in cases with prolonged AXC times and utilising multi-dose strategies varies in the literature [9, 10, 19, 20, 22, 26]. Our study demonstrated no difference in postoperative major adverse events including transfusion, IABP use, maximum inotropic duration, myocardial infarction, acute kidney injury, stroke or mortality. There was no difference in minimum haemoglobin suggesting there is no significant haemodilution with DNC. This was replicated in our sub-group analysis which demonstrated equivalence in outcomes (Table 8), in keeping with a large cohort study sub-analysis done by Koda et al and the recently published prospective RCT by Garcia-Suarez et al. however we did find a higher incidence of stroke with DNC [6, 9]. In contrast, other cohort studies have demonstrated a higher rate of IABP, stroke, and inotropic support and higher peak postoperative creatinine levels in multi-dose DNC [13, 26]. In this report, it is significant that the median number of cardioplegia doses in the 90 min ACX time primary analysis was one therefore inference on multi-dosing may only be based on the sub-analysis.

In the primary analysis the preference for mode of cardioplegia delivery varied among our groups with DNC administered predominantly antegrade (75%) while HKB cardioplegia was delivered by combination antegrade and retrograde delivery in 64% of patients. This variation likely reflects the patient population chosen (those with prolonged cross-clamp times) and the delivery strategy for multiple dose HKB frequently utilising a combined antegrade and retrograde delivery strategy in these patients. The significance of the mode of re-delivery is unclear and given the high variability in existing literature, highlights the need for randomized controlled trials and standardized guidelines given variable clinical implementation. Expectedly the number of cardioplegia doses was 6 (HKB) compared to 1 (DNC) in the primary analysis and 7 (HKB) versus 2 (DNC) in the sensitivity analysis. The maximum ischaemic time was 32 and 98 min in the HKB and DNC groups reflecting our institutions redosing at approximately 30 and 90 min respectively.

The higher rate of return of spontaneous rhythm and lower need for defibrillation on removal of AXC in DNC is well reported [4, 6, 27–29]. Our results showed the DNC cohort was more likely to return to spontaneous rhythm with multi-dose DNC regiments consistent in primary and sub-analyses (Tables 3 and 7) and is consistent with findings our previously published experience [17].

### Quality improvement

There is no clear dosing regimen for extended cross-clamp time and DNC use. Current protocols are based on experience, vary widely and make inter study comparisons challenging. The dosing regimens in the literature range from an initial dose of DNC of 1000–1200 mL with an additional maintenance dose of 300–1000 mL every 60 min after 90 min of cross-clamp time [4–7, 9–11, 17].

Following our initial evaluation of DNC use, adjustments to practice were made and commonly a DNC dosing protocol with initiation with 1000 mL DNC induction dose followed by a further 500 mL DNC at 90 min ischemic time was utilised. Following evaluation of our current data, departmental morbidity and mortality reviews and recent publications discussing redosing and timing of DNC for cases with extended AXC [19, 20], we have developed our current multi-dose DNC dosing protocol.

The current dosing protocol stipulates that when the total ischemic time is expected to be less than 90 min a single 1000 mL induction DNC dose is used. If the ischemic time is expected to exceed 90 min a 1000 mL DNC induction dose is given followed by maintenance doses of 500 mL DNC administered at approximately 60 min intervals thereafter.

### Limitations

This is a retrospective observational study with inherent limitations associated with the study design. Our report represents an evolving clinical practice reinforced by a culture of measurement and review, visible by multiple dosing protocol adjustments over time, with the most recent iteration of the delivery protocol being adopted in 2023. The choice of variables for the propensity matching is an inherent limitation of the statistical methodology, and additionally, we have not performed sensitivity analyses on the route of cardioplegia administration or surgeon performing the procedure. The variation introduced by being unable to propensity match surgeons is an inherent limitation of the retrospective study design. It is important to note the median number of cardioplegia doses in the 90 min ACX time primary analysis was one, therefore inference on multi-dosing is based on the sub-analysis alone. With regards to clinical outcomes one of the major concerns regarding multidose DNC is lidocaine toxicity and its hypothesized results on myocardial contractility and arrhythmia. Arrhythmia and a quantitative reproducible measure of cardiac output such as inotropic requirements or cardiac index post-operatively were not included. Similarly, rates of return to theatre were higher in the DNC cohort however inferences on the manner and cause of this were not included. A further limitation is that we have not reported a 24-hour Troponin value as one of the two hospitals did not collect this time point, and our troponin profiles are not indexed to renal function nor type of procedure which can introduce confounders to their interpretation relative to these variables. Finally, a significant consideration is the learning curve for the operative team in adopting a new cardioplegic strategy, this will inadvertently introduce patient selection bias and additional confounders about timing of redosing, choice of cardioplegic agent, mode, and rate of delivery; which cannot be accounted for in a retrospective study design.

## Conclusion

This is the largest quantitative cohort study on DNC use in patients with prolonged AXC time in Australia or New Zealand and adds significantly to the limited international reports. Del Nido cardioplegia in more complex and longer procedures has demonstrated equivalent myocardial protection compared with multiple doses of HKB cardioplegia. This study is a quality assurance measure driven by clinical experience and our departmental morbidity and mortality review processes. Randomised, multi-centre trials are needed to develop an evidence-based protocol for multi-dose DNC.

## Data Availability

Data use agreements restrict the distribution of raw study-related data files. Requests for data will be reviewed by the ANZCPR Steering Committee.
